# Who Eats Whom? A global food web derived from citizen science

**DOI:** 10.1371/journal.pbio.3003988

**Published:** 2026-09-22

**Authors:** Bradley C. Allf, Aditi Mallavarapu, David W. Kikuchi, Nikhil Vasudeva, Robert R. Dunn

**Affiliations:** 1 Department of Fish, Wildlife, and Conservation Biology, Colorado State University, Fort Collins, Colorado, United States of America; 2 Department of Computer Science, North Carolina State University, Raleigh, North Carolina, United States of America; 3 Department of Integrative Biology, Oregon State University, Corvallis, Oregon, United States of America; 4 Department of Applied Ecology, North Carolina State University, Raleigh, North Carolina, United States of America; 5 Office of University Interdisciplinary Programs, North Carolina State University, Raleigh, North Carolina, United States of America

## Abstract

Citizen science photographs contain a vast and underused record of species interactions. This Community Page introduces Who Eats Whom, an interactive, searchable food web created from photographs uploaded to iNaturalist.

## Teaser

Citizen science contains abundant yet underutilized data about species interactions. We present Who Eats Whom, a database and public engagement tool for searching and visualizing thousands of feeding relationships derived from iNaturalist data.

## The problem with food webs

Ecological communities comprise both individual species and the ‘tangled bank’ of interactions among those species [1]. Making sense of this network is a goal of fundamental importance to ecology [2]. One particularly important type of ecological interaction is feeding, given its role in mediating energy and material flows through ecosystems. Unfortunately, we lack a detailed understanding of the feeding ecology of most species because it is rare to observe these often fleeting, transient events in the field, and alternative methods for gathering feeding data such as stomach content analysis or DNA metabarcoding can be resource-intensive or have certain biases [3]. Much of the data that do exist are dispersed across publications describing standalone interactions or outlining a spatially constrained food web; centralizing these data is a longstanding goal for ecologists [4].

The emergence of massive citizen science datasets is providing a unique and underutilized opportunity to improve and centralize our understanding of what species eat [5]. iNaturalist, one of the largest citizen science databases, contains hundreds of millions of photographs of wildlife compiled by volunteers from around the world [6]. Even if just a small proportion of these photographs show feeding interactions—photographic ‘bycatch’ in a sense—it would represent a substantial addition to ecologists’ empirical knowledge of the global food web, covering a far wider distribution of geography, time, and species diversity than targeted studies ever could. Taking advantage of this potential data source represents an important next step for citizen science [7,8].

## Building Who Eats Whom

**Fig 1:**
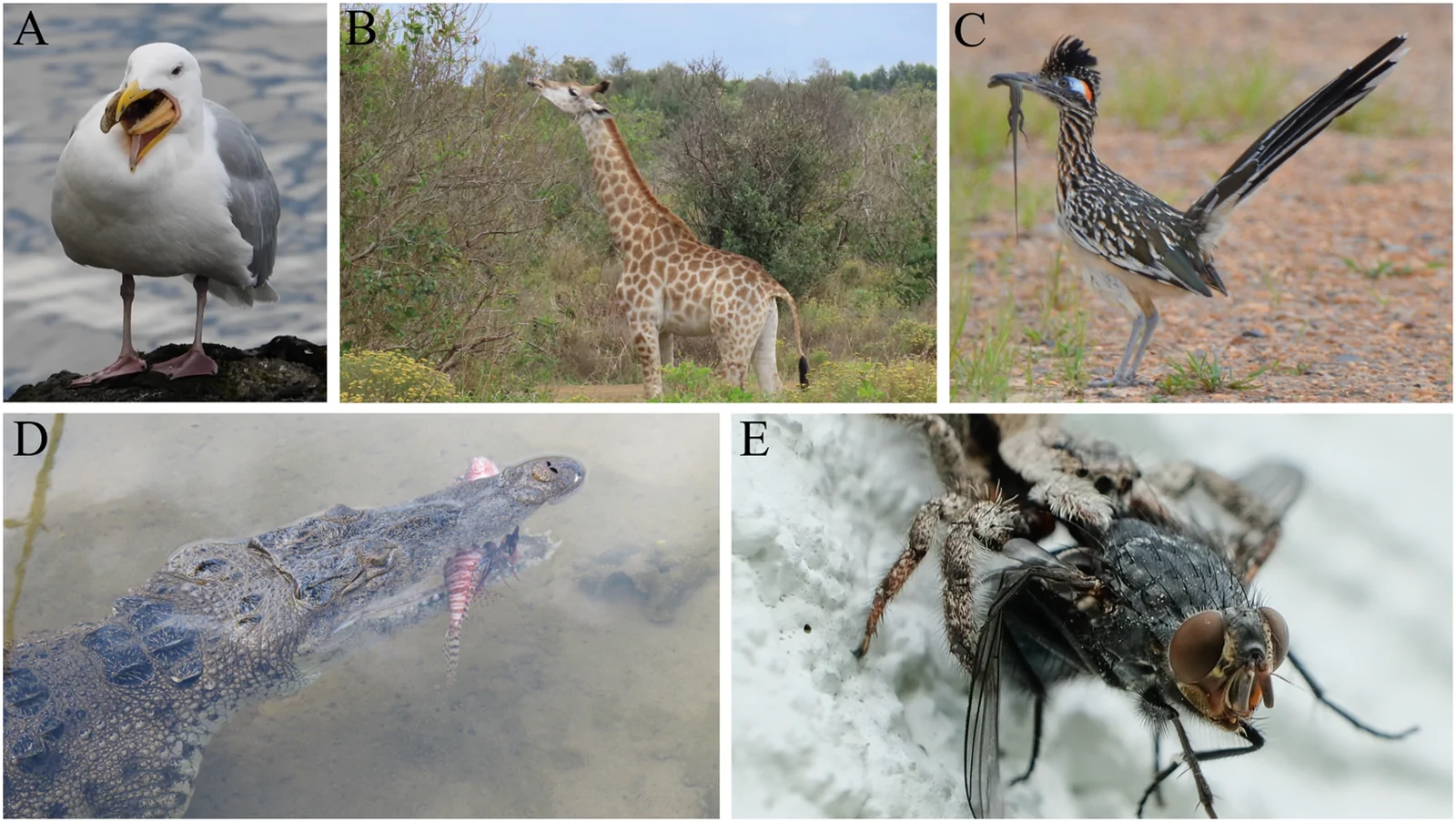
Examples of observations submitted to Who Eats Whom. A. Glaucous-winged Gull (*Larus glaucescens*) feeding on a Mottled Star (*Evasterias troschelii*) by Scott Veirs (PSEMP.net). **B**. South African giraffe (*Giraffa giraffa*) feeding on Duiker-Berry (*Sclerocroton integerrimus*) by MJ Botha. **C**. Greater Roadrunner (*Geococcyx californianus*) feeding on a Six-lined Racerunner (*Aspidoscelis sexlineatus*) by Jody Shugart. **D**. American crocodile (*Crocodylus acutus*) feeding on a Common Lionfish (*Pterois volitans*) by Carlos Domínguez-Rodríguez. **E**. Tan Jumping Spider (*Platycryptus undatus*) feeding on bluebottle fly (*Calliphora* sp.) by Mirko Schoenitz.

Recognizing the potential value of a citizen science-derived database of feeding interactions, we started a project on iNaturalist in 2019 called Who Eats Whom. iNaturalist users participate in the project by submitting observations of wildlife feeding interactions (Fig 1). We define feeding broadly as any interaction in which an organism obtains energy or nutrients by consuming all or part of another organism, encompassing predation and herbivory as well as parasitism, nectar and pollen feeding, and scavenging/decomposition. Participants submit separate observations for both the predator/consumer and the prey/resource. Members of the iNaturalist community propose taxonomic identifications for both observations, from which a community-supported identification is then determined. Users link these observations using metadata tags. Since launching, the project has amassed nearly 14,000 observations of trophic interactions made by nearly 2,000 observers from more than 100 countries. While we have occasionally promoted the project on iNaturalist-related forums, most participation appears to be from volunteers who have discovered the project independently while exploring the iNaturalist database.

In 2025 we began developing a web application for researchers and members of the public to search, visualize, and download Who Eats Whom data, which was released in early 2026. The application leverages real-time (dynamically growing), globally-scoped data from the iNaturalist project collated using an Application Programming Interface. The infrastructure and features are designed to be scalable; given the rapid growth of observations on iNaturalist, it is not unreasonable to imagine a time when there will be hundreds of thousands of interactions included in this initiative. The site (https://whoeatswhom.org/) only uses a subset of high-quality observations from the iNaturalist project, known as ‘research grade’ observations, where there is high community agreement on the taxonomic identity of both species in the observation (importantly, research-grade status does not independently verify that feeding occurred).

**Fig 2:**
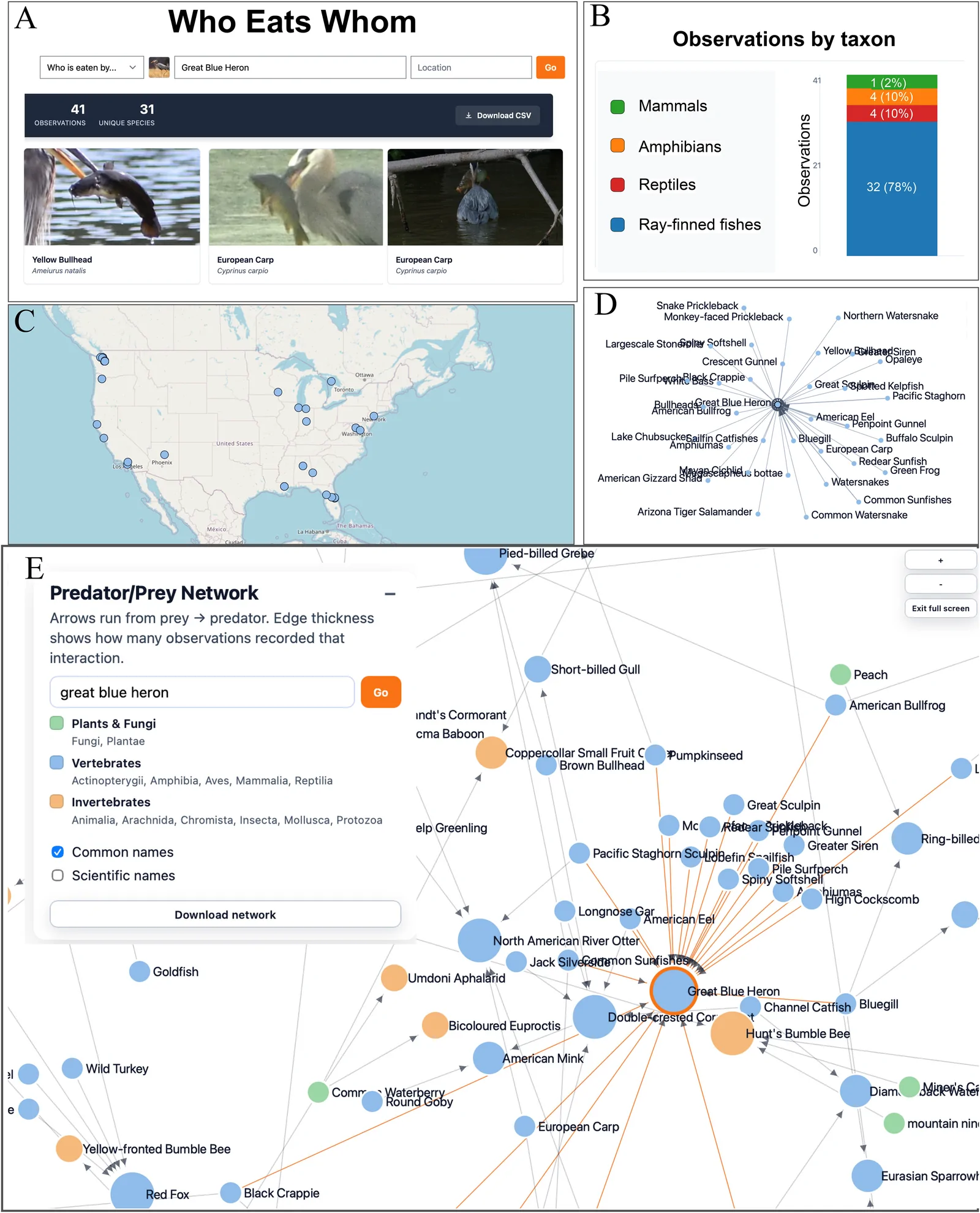
Searching on the Who Eats Whom website. Screenshots from the Who Eats Whom website showing results for the search “Who is eaten by Great Blue Herons” across each tab for visualizing search results: grid view (photos; **A**); graph view (**B**); map view (**C**); network view (a nodal ego network of the interactions involving the searched species; **D**); global view (all nodal interactions among all species in the database; **E**). Note that global view is located in a separate tab called “Interactive Food Web”. Map created with Leaflet using the OpenStreetMap standard tile layer.

Users interact with the site by searching for a species of interest to learn what that species eats, or what eats it and navigating five options for visualizing these results (Fig 2). Users can also download the interactions resulting from their search as a CSV file. In all network visualizations, nodes represent species and directed edges represent feeding interactions, with arrowheads pointing toward the consumer and line widths indicating interaction frequency.

## Uses for Who Eats Whom

Who Eats Whom is, to our knowledge, the first attempt to create a global food web derived specifically from verifiable citizen science photographs [9]. However, there are other emerging databases of species interactions, including ones that draw in part on citizen science data, such as Global Biotic Interactions [10]. We envision Who Eats Whom as a complement to these efforts that also functions as a citizen science project in its own right, engaging volunteers in food web monitoring.

As of January 2026, the Who Eats Whom network contains 1,863 species connected by 1,607 unique interactions. This dataset has the potential to be used by ecologists in several ways, including: searching for basic natural history data about a species’ food needs or natural enemies; finding previously undocumented or rare interactions; identifying highly interconnected prey species that may be particularly important food sources; or identifying data-sparse taxa or regions in need of targeted study. Other potential uses for the data could be analyzing the potential trophic effects of an invasive species, searching for potential biological controls, or exploring the degree of diet specialization versus generalization of a particular species. Cataloguing how diets change across time, season (feeding phenology), life stage (feeding ontogeny) and space/range, and taking stock of what interactions currently exist so they can be conserved could be further avenues for investigation.

Importantly, because Who Eats Whom relies on unstructured (i.e., non-effort-controlled) citizen science data, it necessarily reflects the observational biases of people (e.g., overrepresentation of large, conspicuous, charismatic, or terrestrial interactions). For this reason, Who Eats Whom is best understood as a living natural history archive rather than a complete or representative sample of trophic relationships, and potential applications of the data should be conducted with this in mind. Indeed, even in relatively simple, well-studied systems, food webs are extraordinarily complex and constructing accurate, complete food webs is very challenging [11]. Still, incomplete food webs provide valuable data that can be used for a variety of purposes including sophisticated quantitative methods such as ecological network analysis [2]. Building centralized repositories of feeding interaction data, such as Who Eats Whom, also creates capacity for further merging data collected using diverse and complementary methods to construct networks, from citizen science photos to DNA metabarcoding, allowing for more robust analyses (even if such databases inevitably remain incomplete).

## The future of Who Eats Whom

Beyond our scientific goals, we are designing the project as a tool for education and public engagement in science, given that food webs are a frequent subject in secondary science education courses, and that applied questions about what animals eat may be of broad interest to the public (e.g., “What plants can I grow to support native pollinators?”). With an organized group of volunteers seeking out new observations and reviewing the dataset in real time, Who Eats Whom has the capacity for continued growth and improvement, and being tied to the sophisticated iNaturalist infrastructure for classifying wildlife means the database is constantly updating and improving. Future updates to the site will include improved search queries, improved network visualizations, improved ability to download data, an R package tied to the database, and a system for community vetting of data quality. We are also co-developing guidelines with the iNaturalist community for classifying feeding interactions (parasitism, herbivory, etc.) in the database.

John Muir said of nature, “When we try to pick out anything by itself, we find it hitched to everything else in the Universe” [12]. That meshwork of relationships is just as much a part of the fundamental grandeur of the natural world as species themselves. One could argue that the attention to species and their parts, rather than to the whole, reflects a reductionist turn in Western science. An alternative (or even just a complementary) approach is to focus on the diversity of connections rather than on isolated species. Consider, for instance, the hyper-specialized feeding strategy of caterpillars in the genus *Maculinea*, which mimic ant pheromones and the sounds of ant queens in order to be adopted into an ant colony, where they are fed by worker ants [13]. What makes organisms like *Maculinea* caterpillars inspire wonder is not their mere existence; it is the unique and surprising relationships they have with other species.

Who Eats Whom not only provides a rich and growing dataset for ecological researchers, it also affords the opportunity to bring those data to life through photos and network graphics that allow the public to visualize and tactilely manipulate the complex web of connections that sustains the global ecological system. We anticipate that engaging with this tool could deepen public appreciation of, and interest in, conserving the interconnected natural world.
